# Intelligence

**Published:** 1832-07

**Authors:** 


### INTELLIGENCE.

M. DE BLAINVILLLE has been appointed the successor of CUVIER at the Jardin du Roi. It is probable that M. FLOURENS will succeed that distinguished and lamented philosopher at the College of France. The situation of perpetual secretary to the Académie des Sciences is sought for by the élite of the French savans: M. GEOFFRY SAINT HILIARE, at the last meeting, addressed a letter to the President, stating the claims which he has to this important office.—*Gazette Médicale*.

### CHOLERA.

*Injections of large Quantities of Saline Solutions into the Veins, successfully adopted in some Cases of Malignant Cholera.*

THE facts that have been stated upon this subject are so extraordinary, that, if they did not rest upon the most undoubted authority, we confess we should have been a little sceptical as to their accuracy, and have been induced to suppose, not perhaps that wilful misrepresentation had been resorted to, but that, by some means or other, errors had crept into the account. The respectability and professional talents of the various gentlemen who have recently addressed their brethren upon this subject leave, however, no room for doubt. The facts are certainly as they are stated, however marvellous they may appear. The first communication we give has been transmitted to us by the Central Board of Health: the following is a copy of it.

"Sir: I conceive it to be my duty to let you know, for the information of the Central Board of Health, that the great desideratum of restoring the natural current in the veins and arteries, of improving the colour of the blood, and recovering the function of the lungs in cholera asphyxia, may be accomplished by injecting a weak saline solution into the veins of the patient. To Dr. Thomas Latta, of this place, is due the merit of first having recourse to this practice. He has tried it in six cases; three of which I have seen, and assisted to treat. The most wonderful and satisfactory effect is the immediate consequence of the injection. To produce the effect referred to, a large quantity must be injected, from five to ten pounds in an adult, and repeated at longer or shorter intervals, as the state of the pulse and other symptoms may indicate. Whenever the pulse fails, more fluid ought to be thrown in to produce an effect upon it, without regard to quantity. In one of the cases I have referred to, 120 ounces were injected at once, and repeated to the amount of 330 ounces in twelve hours. In another, 376 ounces were thrown into the veins between Sunday, at eleven A.M., and this day (Tuesday), at four P.M.; that is, in the course of fifty-three hours, upwards of thirty-one pounds!

"The solution that was used consisted of two drachms of muriate, and two scruples of carbonate of soda, to sixty ounces of water. It was at the temperature of 108 or 110 degrees.

"The apparatus employed for injecting was merely one of Reid's common syringes, (the fluid being put into a vessel rather deep and narrow,) with a small pipe fitted, that it might easily be introduced into an incision in the veins of the usual size that is made in bleeding. It may, however, be well to keep in mind that, in the event of the operation being frequently repeated, it may be advisable to inject by different veins.

"I forbear at present to enter further into the particulars, nor have we had sufficient experience to speak decisively on the subject. I may, however, mention that the idea of having recourse to this remedy in cholera occurred to Dr. Latta, from being convinced (which I am also) that the evacuations upwards and downwards are in reality the serum of the blood; that it is the duty of the physician to replace it as speedily as possible, by injecting a fluid as similar to the serum as can be formed artificially, directly into the veins, which has been done here with wonderful and, so far as we can yet judge, excellent effect. An immediate return of the pulse, an improvement in the respiration and in the voice, an evolution of heat, an improvement in the appearance of the patient, with a feeling of comfort, are the immediate effects. The quantity necessary to be injected will probably be found to depend upon the quantity of serum lost, the object of the practice being to place the patient in nearly his ordinary state as to the quantity of blood circulating in the vessels.

I have, &c.

(Signed) ROBERT LEWINS, M.D.
Fellow of the Royal College of Physicians, and
Member of the Leith Board of Health.

"*Leith*, 6, *Quality street; May* 15*th*, 1832.

"To W. MACLEAN, Esq., Secretary to the Central Board of Health.

"Sir: I did myself the honour to address a letter to you lately on the effect of injecting a saline solution into the veins of a patient labouring under cholera. We have not frequent opportunities now of trying this (which I denominate) admirable remedy, as the disease is decidedly less frequent here; but I have seen it employed in two other cases, in the course of the last two days, with the same excellent effect. Sixty ounces are generally thrown in at once, and repeated at the end of three or four hours. In a case to-day, where I saw fifty-eight ounces injected, (being the third time of performing the operation,) the patient's pulse at the commencement was 180, very small, and very feeble; she was excessively restless, with a feeling of great weakness and tormenting thirst. Before twelve ounces were injected, the pulse began to improve; it became fuller and slower, and it continued to improve until, after fifty-eight ounces had been injected, it was down to 110. Before I left the patient (a woman) her condition was altogether amazingly ameliorated. There was a fine glow and a slight perspiration on her face; the veins on the back of her hand were well filled; the restlessness was removed; and the feeling of excessive weakness gone, and the thirst ceased. The pulse was under 100, and free, full, and soft! Verily, sir, this is an astonishing method of medication, and I predict will lead to wonderful changes and improvements in the practice of medicine. I have addressed you upon the subject as the organ, from your high official station, of most speedily and effectually disseminating a knowledge of the extraordinary facts referred to. It will, of course, give me great pleasure to enter further into particulars upon any particular point on which you may require information in reference to the cases that have come under my observation.

"I have, &c.

(Signed) "ROBERT LEWINS.

"6, *Quality street, Leith; May* 18, 1832.

"To W. MACLEAN, Esq., Secretary to the Central Board of Health.

"In the hands of a man of ordinary dexterity, the common injecting apparatus alluded to in my last will be found to answer the purpose perfectly well; but if the practice I recommend is (as I hope it will be) generally adopted, it will, I conceive, be expedient to advise that a regular and perfect transfusion apparatus be used; at all events, to warn those who inject to beware of allowing air to get into the vein. The tubes, of course, must be filled with fluid, as well as the pipe in the vein, before commencing, and considerably more fluid than it is intended to use ought to be in the vessel from which it is pumped.

"R. L."

In consequence of the receipt of the above, the Central Board of Health put various queries to Dr. Lewins relative to saline injections into the veins in cholera, to which the following replies were given:

| QUERIES. | ANSWERS. |
|---|---|
| 1. Were any of your patients bled previously to, or after, the saline injections into their veins? | 1. None before; one to the amount of twelve ounces immediately after the first injection. |
| 2. Were the evacuations by purging, vomiting, or perspiration, increased by the injections? | 2. The evacuations by purging and vomiting, in most of the cases, continued. In some of them, the purging, the discharge from the bowels at least, was increased. Perspiration was increased in all. |
| 3. Did any of the patients submitted to the saline-injection plan die; and, if examined after death, what were the appearances? | 3. Yes; no less than ten of the fifteen that have been injected up to the present day, but under such circumstances as do not detract from the general merits of the practice. This will be made evident by the history of the cases that will be sent by to-morrow's post. |
| 4. Had the pulse at the wrist absolutely ceased, and for how long; or had blueness of the surface taken place, and to what extent, in any of your patients, before the injection of the saline fluids: and how many of such patients recovered under that treatment? | 4. Yes, and for hours, even at the axilla: in some of the cases, blueness of the surface had taken place to a considerable extent. Five of these patients recovered. |
| 5. Had suppression of urine been perfectly established, and for how long, in any of your cases previously to the saline injection; and what effect did that practice appear to produce on the urinary secretion? | 5. Complete suppression, I think, in all, except two, and for hours. In all the successful cases, the effects of the injection in restoring the secretion of urine was most evident. |
| 6. What effect did the injections appear to have on the temperature of the patient? | 6. The injections raised the temperature of the body; but in all the successful cases, when the veins were injected, the patient complained of cold soon after the injection. |
| 7. Were the blood and evacuations analysed before and after the injection? | 7. Neither the blood nor the evacuations were analyzed; but I sent some of the blood of a patient that had been injected by the veins to Dr. Reid, for analysis to-day. |
| 8. Did consecutive fever occur in any, and, if so, in how many of your cases, whether successful or otherwise? | 8. The consecutive fever, in all the patients who were injected, has been slight. |
| 9. Was the quantity of the evacuations noted before and after the Injections in any of your cases? | 9. No; but they were excessive in most of the cases. |
| 10. Please to give the details of two or three cases treated by saline injections, with age, condition of life, temperament, habits, &c.; and particulars of such other treatment as may have been adopted, in addition to the saline injections. | 10. Question ten shall be fully answered by to-morrow's post. |

(Signed)
Robert Lewins, M.D.

6, *Quality street;*
*May* 26*th, two o'clock* A.M.

In the "Lancet" of June 2d is a letter from Dr. Latta to the Secretary of the Central Board of Health, London, affording a view of the rationale and results of his practice in the treatment of cholera by aqueous and saline injections. Dr. Latta states that, although as yet his experience of this mode of treatment is limited, he thinks he can adduce sufficient proof, not only of its safety, but of its unquestionable utility. He has never yet seen one bad symptom attributable to it, and he has no doubt it will be found, when judiciously applied, to be one of the most powerful and one of the safest remedies yet used in the second stage of cholera, "or that hopeless state of collapse to which the system is reduced." The plan was suggested to Dr. L. by reading in the Lancet the review of Dr. O'Shaughnessy's Report* on the Chemical Pathology of Malignant Cholera, by which it appears that in that disease there is a very great deficiency both of the water and saline matter of the blood. Dr. Latta first attempted to restore the blood to its natural state by copious saline injections thrown into the large intestines; but as, from this plan, the vomiting, tormina, and purging were much aggravated, he resolved to throw the fluid immediately into the circulation. In this, having no precedent to direct him, he proceeded with much caution. The first subject of experiment was an aged female, on whom all the usual remedies had been fully tried, without benefit.

"She had apparently reached the last moments of her earthly existence, and now nothing could injure her; indeed, so entirely was she reduced, that I feared I should be unable to get my apparatus ready ere she expired. Having inserted a tube into the basilic vein, cautiously, anxiously, I watched the effects: ounce after ounce was injected, but no visible change was produced. Still persevering, I thought she began to breathe less laboriously: soon the sharpened features, and sunken eye, and fallen jaw, pale and cold, bearing the manifest impress of death's signet, began to glow with returning animation; the pulse, which had long ceased, returned to the wrist; at first small and quick, by degrees it became more and more distinct, fuller, slower, and firmer, and in the short space of half an hour, when six pints had been injected, she expressed in a firm voice that she was free from all uneasiness, actually became jocular, and fancied all she needed was a little sleep; her extremities were warm, and every feature bore the aspect of comfort and health. This being my first case, I fancied my patient secure, and from my great need of a little repose, left her in charge of the hospital surgeon; but I had not been long gone, ere the vomiting and purging recurring, soon reduced her to her former state of debility. I was not apprised of the event, and she sunk in five and a half hours after I left her. As she had previously been of a sound constitution, I have no doubt the case would have issued in complete reaction, had the remedy, which already had produced such effect, been repeated."

Dr. Latta dissolved from two to three drachms of muriate of soda and two scruples of the subcarbonate of soda in six pints of water, and injected it at a temperature of 112° Fahr. If the temperature is as low as 100, it produces an extreme sense of cold, with rigors; and if it reaches 115°, it suddenly excites the heart, the countenance becomes flushed, and the patient complains of great weakness.

"At first there is but little felt by the patient, and symptoms continue unaltered, until the blood, mingled with the injected liquid, becomes warm and fluid; the improvement in the pulse and countenance is almost simultaneous, the cadaverous expression gradually gives place to appearances of returning animation, the horrid oppression at the præcordia goes off, the sunken turned-up eye, half covered by the palpebræ, becomes gradually fuller, till it sparkles with the brilliancy of health, the livid hue disappears, the warmth of the body returns, and it regains its natural colour; words are no more uttered in whispers, the voice

* For an analysis of this work, see London Med. and Phys. Journal, May 1832, p. 389.—Editors.

first acquires its true cholera tone, and ultimately its wonted energy, and the poor patient, who but a few minutes before was oppressed with sickness, vomiting, and burning thirst, is suddenly relieved from every distressing symptom: blood now drawn exhibits, on exposure to air, its natural florid hue."

Notwithstanding the appearances of these favorable symptoms, Dr. Latta remarks that the utmost vigilance is still necessary. At first the change is so great, that the physician may fancy all is accomplished; but the diarrhœa recurring, he may find the patient, after the lapse of two or three hours, as low as ever. As soon as reaction by the first injection is produced, mild warm stimulants, such as weak gin toddy, mixed with some astringent, should, he says, be freely and assiduously administered.

"An attempt should be made to fill the colon with some astringent fluid. That such is requisite, is evident from the watery diarrhœa returning with violence, and if not restrained, death will ultimately make sure of his victim: therefore so soon as the pulse fails, and the features again shrink, the venous injection must be repeated, taking care that the fluid in use retains its proper temperature. The injection should be carried on very slowly, unless the patient is much exhausted, when it may be used more rapidly at first, until a little excitement is produced, after which it should not exceed two or three ounces per minute; and now is the time for the exhibition of astringents by the mouth, which will be retained, for in general the sickness entirely leaves during the operation.

"Such remedies must be persisted in, and repeated as symptoms demand, or until reaction is permanently established. I have witnessed no violent symptoms accompanying the rapid injection of the fluid, but I have thought that the hasty repletion of the system was followed by great increase of the evacuations, and consequently a more sudden depression of the powers of life. The quantity to be injected depends on the effect produced, and the repetition on the demands of the system, which generally vary acccrding to the violence of the diarrhœa; the greater the degree of collapse, the greater will be the quantity needed, though not uniformly, for a very slight loss produces much depression in some systems: hence there is often great collapse, without much vomiting, purging, or cutaneous discharge.

"Although in every case, even the most desperate, the cholera symptoms were removed, some of my cases failed, which I attributed to one or other of the following causes: either the quantity injected was too small, or its effects were rendered abortive by extensive organic disease, or its application was too lae.

"I have already given an instance where deficiency in quantity was the cause of failure, which I will now contrast with one in which it was used freely. A female, aged fifty, very destitute, but previously in good health, was on the 13th instant, at four A.M., seized with cholera in its most violent form, and by half-past nine was reduced to a most hopeless state. The pulse was quite gone, even in the axilla, and strength so much exhausted, that I had resolved not to try the effects of the injection, conceiving the poor woman's case to be hopeless, and that the failure of the experiment might afford the prejudiced and illiberal an opportunity to stigmatize the practice: however, I at length thought I would give her a chance, and in the presence of Drs. Lewins and Craigie, and Messrs. Sibson and Paterson, I injected one hundred and twenty ounces, when, like the effects of magic, instead of the pallid aspect of one whom death had sealed as his own, the vital tide was restored, and life and vivacity returned; but diarrhœa recurred, and in three hours she again sunk. One hundred and twenty ounces more were injected with the same good effect. In this case 330 ounces were so used in twelve hours, when reaction was completely reestablished; and in forty-eight hours she smoked her pipe free from distemper. She was then, for better accommodation, carried to the hospital, where, probably from contagion, slight typhoid symptoms were produced. She is now, however, convalescent.

"The second cause of want of success is the presence of organic disease: this, probably, renders the possessor very liable to attacks of cholera; and the latent evil, which previously gave but little uneasiness, suffers aggravation in all its symptoms, more especially after reaction has been produced, and has evidently, in many cases, been the cause of death. A delicate young female, of strumous habit, who had been for some years subject to pectoral complaints, was rescued from a state of collapse by the injection of sixty ounces of the saline fluid, administered in separate portions, within the space of twelve hours. After lingering for ten days she died; the heart was found in a state of atrophy, covered with strong evidence of the existence of ancient disease, and floating in eight ounces of pus. In another case every internal organ was diseased; some of them so much so, that it was astonishing the individual lived so long.

"The third cause of the occasional want of success, is the late application of the remedy. Hitherto I have had opportunity of injecting only in extreme cases, after every other means had entirely failed, cases which apparently would have soon proved fatal. Here the obstacles to be overcome have been of no ordinary kind, notwithstanding the result of the practice is of the most encouraging nature, and the number of cases now convalescent or doing well highly gratifying. In every fatal case we have had an opportunity of examining, independent of organic disease, I have found a large quantity of fibrin in the cavities of the heart, especially on the right side, where it had extended from the auricle through the ventricle into the pulmonary artery. Such deposition must have formed a certain obstacle to recovery, and is, no doubt, from the interruption it gives to the pulmonary circulation, the cause of the heavings of the chest, and the inordinate action perceptible in the centre of circulation many hours before death. Now surely it is reasonable to suppose, that if this, the most simple of all remedies, were applied early, before the blood, drained of its water has collected in the larger vessels, in fact before such fibrinous depositions have taken place in the cavities of the heart, is it not reasonable to suppose that such would be entirely prevented?

"But not only is early injection advisable on this account, not only is stagnation of the blood prevented by it, and the laborious breathing, and the præcordial oppression, the intense sickness, the burning thirst, the extreme depression of the vital powers, and the chances of aggravating chronic disease, or of producing new organic lesion, in a great measure avoided, but it is rational to suppose that the consecutive fever will be rendered much milder; and that this is the case, is supported by my own experience, even though the remedy has not been applied earlier; indeed the fact is very evident. In an ordinary attack of cholera much fluid is lost; and if the individual is so fortunate as to get out of the stage of collapse, if consecutive fever of typhoid type comes on, the system, left to its own resources to replace the lost serum, must be but ill fitted for the task; for the debility is extreme, absorption goes on slowly, the fever will be much aggravated by the irritation of internal congestion; local inflammation will thereby be produced, and the chance of recovery will be but small. Much of this evil is to be mitigated or entirely avoided by injection into the veins, of which circumstance I can adduce living instances; and where the patient who had been injected has sunk under organic disease, the usual marks of congestion are not perceptible.

"The apparatus I have used is Read's patent syringe, having a small silver tube attached to the extremity of the flexible injecting tube. The syringe must be quite perfect, so as to avoid the risk of injecting air; the saline fluid should never be injected oftener than once into the same orifice, and the vein should be treated with much delicacy, to avoid phlebitis. The wound should be poulticed and carefully watched, if it does not heal by the first intention."

In a second letter from Dr. Lewins, he states that in the Drummond-street Hospital, Edinburgh, six patients have been injected, and three recovered or are recovering. In the three that died, extensive organic disease was found on dissection; disease that had existed previously to the attack of cholera.

Dr. Craigie, of Leith, has also recorded two cases of malignant cholera which he treated by venous injection. The first case was successful, and in it fifteen pounds were injected, at intervals, in nine hours. In the second case, the patient appeared likely to die within an hour or two. The injections, however, afforded her some relief for a few hours.

In another communication from Dr. Lewins to the Central Board of Health, he mentions the following case that occurred at the Leith Cholera Hospital, on May 28th.

"A woman, of about forty years of age, was admitted on Sunday evening at seven o'clock. She was pulseless even at the axilla, sightless, cold and blue over almost the whole body. Respiration very slow and irregular; in a word, she was all but lifeless. It was feared she would be dead before the operation of injecting could be commenced. Between seven at night and two o'clock next morning, there were thrown in 284 ounces, upwards of twenty-three pounds. The report of her situation at two o'clock on Monday morning, in the hospital book, is as follows: 'A change for the better that appears almost miraculous has taken place. The action of the heart is greatly improved; respiration not in the least laborious, but quicker than natural; pulse 120, small, but distinct. She can articulate distinctly; countenance natural; lips red; tongue moist and warm; she perspires freely; heat over the whole body natural.'"

Some very interesting cases have also been published by Mr. Alexander Tweedie, in the Medical Gazette, June 9th, in which this treatment was adopted. In the first case, *nearly a gallon* of saline injection was thrown into the veins, with temporary improvement, but ultimate failure. In the second case, the beneficial effects of the treatment were but temporary. In the third, it was successful. In the fourth, saline injection into the veins was adopted, with temporary improvement, followed by relapse and death.

Dr. Hope has also related two cases in the Medical Gazette of June 16. In the first, eight pints were injected in three hours, with remarkable temporary benefit, but ultimate failure. In the second, seven pints were injected at three times in five hours. Calomel and opium were also given. The patient recovered.

As we are desirous to communicate every particular relating to this novel and curious mode of treatment, we subjoin some remarks made by Dr. O'Shaughnessy in a letter in the Lancet of June 2d.

"I write now for the purpose of offering the following suggestions to the gentlemen engaged in these experiments.

"Although by the injection of water and salts (in quantities varying according to the previous extent of the evacuations), we may restore the deficient fluids of the body, and bring back the blood to its normal state, thus possibly removing a powerful cause of death in this disease, we must still remember that the unknown remote cause, and other agents, may be and still are in operation, and require to be remedied before a perfect cure can be performed. The great debility, the natural effect of the evacuations, will probably require some stronger stimulant than the new and artificial blood will afford. Some means will also have to be taken to prevent the recurrence or continuance of those evacuations, an event of constant occurrence in Dr. Latta's cases.

"I would therefore recommend that, in addition to the injection of water, either simultaneously or subsequently, as circumstances would suggest to the operator, medicinal agents, of the stimulant or astringent classes, for example, should also be injected in minute doses.

"In selecting the agents employed, it is to be cautiously observed that they must not be chemically incompatible with the blood. They must not coagulate albumen, fibrin, or colouring matter, or darken the colour of the latter. As a general rule, they should be soluble in water.

"As stimulants, minute quantities of carbonate of ammonia dissolved in water might be used. Dupuy, of Alfort, has found that this salt may be injected with safety into the veins of horses. Small doses of the sulphate of quinine might also be thus administered. Even extremely dilute spirits might be employed.

"Weak decoctions, or solution of the vegetable astringents, might also, I conceive, be injected with great advantage. Gaspard's experiments fully warrant me in stating that these cannot induce any bad effect, and that they may therefore be tried with perfect safety."

We regret that our limits prevent us from giving the details of the various cases above referred to. In some of them, in which the patients were considered in an utterly hopeless state, the treatment by injection was followed by recovery; while in many of the instances in which it failed, such extensive organic disease existed, that permanent benefit could not be expected from any medical agency. In every point of view, the subject is one of the deepest interest, and we shall not fail to collect any additional facts that may hereafter be promulgated concerning it.

---

### OFFICIAL PAPER ON CHOLERA.

*Council Office, Central Board of Health;*
*9th May,* 1832.

Precautionary Hints *to Persons residing in Places suffering or likely to suffer from* Cholera; *with* Concise Directions *for the Treatment of those threatened with, or actually attacked by, the Disease, in Situations where Medical Advice cannot be immediately obtained.*

Heads of families living in the country, and benevolent individuals wishing to afford remedial assistance in this destructive malady, ought to provide themselves with the following articles, viz.

Tincture of Opium (Laudanum), 2 oz.; Tincture of Catechu, 4 oz.; Tincture of Assafœtida, 4 oz.; Aromatic Spirit of Ammonia, 4 oz.; Compound Spirit of Lavender, 2 oz.; Oil of Peppermint, half an ounce; Castor Oil, 2 lb.; Ipecacuanha in powder, 2 oz.; Mustard in ditto (best Durham), 10 lb.; Compound Chalk Powder, 4 oz.; Sulphate of Quinine, 1 oz.

Pills, No. 1. Six dozen; Calomel, two and a half grains, Opium, a quarter grain, and Cayenne Pepper, two grains, in each pill.

No. 2. Three dozen; Calomel and Compound Extract of Colocynth, of each two and a half grains in each pill.

No. 3. Three dozen; Bluepill and Rhubarb, of each two grains in each pill.

Powders, No 4. Calcined Magnesia, two parts, Rhubarb in powder, two parts, Ginger in ditto, one part, carefully mixed; one ounce.

No. 5. Calomel, one grain, James's Powder, two grains, and Nitre in powder, five grains; half an ounce.

Liniment, No. 6. Compound Soap Liniment with Opium, eight parts, Tincture of Cantharides, one part; three ounces.

Mustard Poultice, No. 7. The mustard poultice is made by mixing equal parts of mustard powder and crum of bread into a paste with hot water; or by mixing equal parts of mustard powder and thick porridge.

Bags or stockings, to hold heated bran or salt; stomach and feet warmers; enema syringe; a graduated glass measure, (one ounce;) a set of scales and weights, (grain.)

The above supply is calculated for the number likely to be attacked in a population of five hundred; and in price, as estimated by a London chemist, will not exceed 3*l.* 3*s.*

*Precautions.* 1. *The clothing* should be warm. Woollen stockings ought to be worn, and flannel next the skin, at least over the belly and loins.

2. *Diet.* Avoid, above all things, overloading the stomach: indigestion, however produced, disposes the body to this disease. If in easy circumstances, take for dinner a moderate quantity of roast meat in preference to boiled, with stale bread or good potato, two glasses of wine with water, or an equivalent of weak brandy or whisky and water, or of sound porter or ale. Eat garden stuff and fruit sparingly, and avoid fat luscious meats. In short, whilst under apprehension of cholera, use a dry, nutritive diet, sparing rather than abundant; observe great caution as to eating suppers, for cholera most frequently attacks about midnight, or very early in the morning.

In case of costiveness, take one or two of the pills No. 3, going to bed; or one or two of the pills No. 2, in the morning, should no effect be produced by No. 3; but to avoid salts, senna, and all cold drastic purgatives.

3. *Exercise.* Moderate exercise in the open air, in fine weather, is conducive to health; but the greatest care should be observed by all, more especially by the weakly and the aged, not to carry that exercise to fatigue or profuse perspiration, nor to sit down with wet feet or wet clothes.

*Treatment of the Premonitory Symptoms of Cholera.* 4. In a very large majority of cases, the attack of cholera is preceded by a looseness of the bowels, of longer or shorter duration, say twenty-four hours. It is in this stage that remedial assistance is most efficient, and that life may be saved with the most certainty, by checking the disease in its commencement. When, therefore, the bowels become relaxed without an obvious cause, where cholera is prevailing at the time, the following measures should be adopted without loss of time:

5. In the case of adults, previously healthy, let blood be taken from the arm to eight or ten ounces, or by ten or twelve leeches to the pit of the stomach, or by cupping.

Should the loose motions be of a darker colour than natural, give two pills of form No. 2, and four hours after a tablespoonful of castor oil, floating on half a wine-glassful of gin and water, brandy and water, or cold coffee, with ten drops of laudanum if there be griping pains. Confine the patient strictly to bed, and give the following draught at night: Cinnamon or peppermint water, half an ounce;* Laudanum, twenty-five drops.

6. When the purging is of the ordinary, bilious, and feculent kind, with griping and flatulence, give ten drops of laudanum and forty of tincture of catechu in the same vehicle, every hour, for five or six hours; or twenty grains of the compound chalk powder every second or third hour, should relief not be obtained sooner.

A warm bath for half an hour, followed by rubbing with flannel or flesh brushes; warm fomentations to the belly, by means of bladders half filled with hot water, or flannels soaked in hot spiced wine, or in hot spirit and water, will afford much relief.

7. When there are cramps, a desert-spoonful or two of the liniment No. 6 should be assiduously rubbed on the part affected.

8. If there be nausea or sickness, without acute pain at the pit of the stomach, give an emetic of twenty-five or thirty grains of ipecacuanha in half a pint of warm water.

9. When giddiness and pain at the pit of the stomach are present, bleed as above, and give a teaspoonful of the aperient powder No. 4.

10. Let the diet, in all these premonitory stages, consist of light farinaceous preparations: sago, tapioca, panada; chicken broth, and tepid drinks to promote perspiration.

11. Should debility, with chills and sweats, remain, give two grains of sulphate of quinine three times a day, for two or three days. This medicine will often be found to check the relaxation of the bowels.

*First Stage of the Attack. Treatment.* 12. When the motions have lost the appearance of feculent matter, and have put on that of rice water or chicken broth, with vomiting of similar liquids, spasms, intense thirst, irregular, slow, and weak pulse, give an emetic of half a pint of a solution of common salt, as strong as it can be made, with a teaspoonful of mustard powder. Place a mustard poultice, No. 7, over the whole stomach, belly, and front of the short ribs, having previously rubbed the parts with the liniment. Give one of the pills No. 1 every alternate half hour, and in the intervals two table-spoonsful of weak brandy or whisky and water; cold, if preferred. Let the patient drink cold water, or iced water if it can be had, allowing no more than two or three table-spoonsful at a time; or bits of ice the size of a nut may be given to be swallowed whole, to allay the burning sensation at the pit of the stomach. Let bags or stockings, filled with heated bran or sand, be placed along the patient's spine or sides, and feet warmers applied to his feet. Let him be kept still, if possible, wrapt in warm blankets, but not oppressed with heat or coverings, particularly over the chest and neck.

*Second Stage of the Attack.* 13. If, notwithstanding these measures, the patient should appear to be sinking, the pulse becoming weaker, the skin colder, the breathing more laborious, the individual appearing less anxious about his own situation, then, in addition to the steady application of the measures already recommended, let an injection be thrown up the rectum, consisting of two or three pints of water, as warm as the hand can conveniently bear, with a small wine-glassful of brandy or whisky, to be repeated, if thought necessary, at intervals of an hour.

*Third Stage.* 14. When the pulse at the wrist has ceased, or become almost imperceptible, with coldness of the extremities, and perhaps blueness of the surface, particularly of the lips, hands, and feet; irregular breathing, loss of voice, suppression of urine, ghastly counte-

* Peppermint water may be made by rubbing down five drops of oil of peppermint with half a teaspoonful of sugar, adding a tablespoonful of water by degrees.

nance, without delirium; although, under these awful circumstances, there is but little room for hope, our exertions should not cease.

15. At this stage of the attack, the vomiting and purging will generally have ceased, or at least be much diminished; the belly will be drawn in, and pain, sinking, and death-like oppression, will be felt about the heart.

16. Let the hot-water injection be repeated, with two or three drachms of the tincture of assafœtida, and retained for some minutes by means of a napkin.

17. Let mustard poultices be applied to the inside of the thighs and calves of the legs, in addition to that on the belly, which may be removed to the sides of the chest or back; let the limbs be diligently rubbed with warm cloths; let small quantities of light cordials be given at intervals, such as a teaspoonful of compound tincture of cinnamon, or of aromatic spirit of ammonia, in a table-spoonful of water, and let the treatment ordered for the second stage be continued until the pulse becomes distinctly perceptible at the wrist.*

*Stage of Reaction, or Fever.* 18. When the pulse has begun to rise, and the heat and natural colour begin to return to the surface, keep the patient perfectly quiet, but let him be carefully watched, for a sudden sinking of the powers of life not unfrequently occurs at this period of the disease. Opiates of all kinds must now be withheld; and wine, brandy, and other stimulants used very sparingly, and withdrawn altogether as soon as the pulse and heat are steadily re-established; when mild tepid drinks are to be substituted, and the powder No. 5 given every hour, instead of the medicines hitherto used, should the bowels be torpid.

19. Under this treatment, a warm copious sweat often breaks out, or a more healthy discharge takes place from the bowels, or some urine is passed, which, of all others, is the most favorable sign. When such is the case, the patient, with proper care, will often pass into a state of convalescence, without further difficulty or danger.

20. It often happens, however, notwithstanding all our care, that the re-establishment of the pulse and heat are closely followed by symptoms of fever, or by some degree of stupor, or by great oppression of breathing, or by distention and tenderness of the belly; all of which indicate danger.

21. The moment such symptoms appear, bleed from the arm, or from the part most affected, by leeches or cupping, to ten, twelve, or sixteen ounces, according to the effect produced by the bleeding. Reduce the temperature of the patient's room, give cool drinks, and apply cold wet cloths or pounded ice in bladders to the head; and give the powders No. 5, as already ordered.

22. When convalescence has begun, observe the strictest care as to diet. At this period a full meal has, in numerous instances, brought on a relapse: indeed, animal food, even in small quantity, under these critical circumstances, has often been attended with dangerous consequences to those just recovering from cholera. To such, even the mildest articles of food should be given in much smaller quantities and at shorter intervals than to those in health; and their ordinary diet and habits should be very cautiously resumed.

W. PYM, Chairman.

### LITERARY INTELLIGENCE.

(In the Press.) Cheap and accurate Figures of the British Flowering Plants found in Oxfordshire and its contiguous Counties, and described in WALKER's Flora. Drawn from Nature, and engraved under the Direction of Mr. WM. BAXTER, A.L.S. F.H.S. &c., Curator of the Oxford Botanic Garden.

The object proposed in the present work is to supply the lovers of botany with a set of figures, which may be relied on for accuracy, while every unnecessary expense will be avoided. With this view, it was at first proposed to reduce the size of the plants on a plan similar to that in "Maund's Botanic Garden;" but this was not found compatible with sufficient distinctness and accuracy.

The work will be proceeded with so soon as a sufficient number of subscribers' names to give a reasonable prospect of success shall have been received.

It is proposed to divide the work into three series: the first to consist of one plate to each genus, as a guide to students; the second to comprise the re-

* The following plan of treatment, proposed by Dr. Stevens, and acted upon under his direction, has excited some notice, and is stated to have been attended with very considerable success in all stages of the disease: Supercarbonate of Soda, half a drachm; Muriate of Soda (common salt), twenty grains; Chlorate of Potass, seven grains. To be given in half a tumbler of water every hour, until the patient begins to recover from the collapse.

Dry heat, frictions, mustard poultices, and injections of hot salt and water were used at the same time.

mainder of the Oxfordshire flowering plants; and the third (if called for) to complete the British Flora.

No. I. contains

Plate I. FRITILLARIA MELEAGRIS.—Fritillary. Chequered Daffodil. Snake s head.

Linn. Class 6. Hexandria. Order 1. Monogynia.

Natural Order, Liliaceæ. Jussieu. Lindley's Synopsis, p. 266.

Localities: Moist meadows and pastures; Magdalen College Meadow; Cowley Meadow; Standlake, Rev. A. M. White; Wroxall Field, Warwickshire, Mr. Perry.

Perennial. April and May.

Fig. 1. A petal, to show the Nectary; fig. 2. Stamens; Fig. 3. Germen, Style, and Stigma

Plate II. TULIPA SYLVESTRIS. Wild Tulip.

Linn. Class 6. Hexandria. Order 1. Monogynia.

Natural Order, Liliaceæ. Jussieu. Lindley's Synopsis, p. 266.

Localities: In old chalk pits in some parts of Suffolk, Norfolk, Herefordshire, and other places; by the side of the walk round Christ-Church Meadow, Oxford; about Allesley; and in meadows by the Bourne at Shustock, Warwickshire, Rev. W. T. Bree; Bitton Meadows. Glocestershire, Rev. H. T. Ellicombe.

Perennial. April and May.

Fig. 1. Stamens; Fig. 2. Germen; Fig. 3. Section of ditto.

*** We have seen proofs of the two first numbers of this work, and have much pleasure in recommending it to the patronage of our readers as the best and cheapest pictorial Flora of the day; and, from the well known skill and science of Mr. Baxter, we doubt not that the characters and dissections will be far superior to any which have yet appeared.

---

### MONTHLY LIST OF MEDICAL BOOKS.

### METEOROLOGICAL JOURNAL,

By Messrs. HARRIS and Co. Mathematical Instrument Makers, 50, High Holborn.

| | Moon. | Rain gauge. | Thermometer. | | | Barometer. | | De Luc's Hygrometer. | | Winds. | | Atmospheric Variation. | | |
|---|---|---|---|---|---|---|---|---|---|---|---|---|---|---|
| *May* | | | 9a.m. | max. | min. | 9a.m. | 10p.m. | 9a.m. | 10p.m. | 9 a.m. | 10p.m. | 9 a.m. | 2 p.m. | 10 p.m. |
| 20 | | | 52 | 59 | 49 | 30.00 | 30.02 | 46 | 48 | E | E | fine | fine | fine |
| 21 | | | 61 | 64 | 53 | .02 | .02 | 49 | 49 | SE | SSW | | | cloudy |
| 22 | ☾ | | 50 | 67 | 54 | .02 | .00 | 49 | 48 | W | NW | | | fine |
| 23 | | | 60 | 68 | 53 | .05 | .06 | 48 | 50 | NNE | NE | | | |
| 24 | | | 61 | 67 | 54 | .07 | .02 | 51 | 50 | SE | SW | | | |
| 25 | | | 53 | 68 | 57 | 29.96 | 29.88 | 50 | 49 | WNW | NNE | | | |
| 26 | | | 65 | 69 | 54 | .77 | .76 | 48 | 48 | NNW | NE | cloudy | | cloudy |
| 27 | | | 63 | 70 | 50 | .76 | .73 | 48 | 48 | N | NE | fine | | fine |
| 28 | | | 62 | 66 | 55 | .72 | .68 | 47 | 49 | WSW | WSW | | | |
| 29 | ● | | 58 | 68 | 50 | .68 | .71 | 49 | 50 | SW | SW | rain | cloudy | |
| 30 | | | 56 | 65 | 51 | .60 | .53 | 50 | 51 | SSW | SSW | fine | fine | rain |
| 31 | | .47 | 58 | 62 | 52 | .29 | .33 | 53 | 56 | SSW | SW | | | |
| *June* | | | | | | | | | | | | | | |
| 1 | | | 55 | 58 | 53 | .40 | .51 | 58 | 58 | S | SSW | rain | showery | cloudy |
| 2 | | | 60 | 68 | 58 | .63 | .63 | 53 | 50 | WNW | ESE | | fine | fine |
| 3 | | | 64 | 71 | 55 | .58 | .41 | 50 | 53 | SW | SW | fine | | |
| 4 | | .23 | 62 | 64 | 54 | .32 | .38 | 55 | 57 | SE | SSE | | | cloudy |
| 5 | ☽ | | 61 | 68 | 54 | .32 | .35 | 57 | 57 | SW | SSW | | showery | |
| 6 | | .22 | 63 | 66 | 53 | .26 | .29 | 56 | 53 | SSE | SW | cloudy | fine | fine |
| 7 | | | 58 | 60 | 52 | .37 | .46 | 53 | 57 | SW | SSW | | | |
| 8 | | .30 | 56 | 63 | 56 | .48 | .58 | 59 | 58 | SE | SSW | | showery | rain |
| 9 | | | 61 | 66 | 53 | .61 | .63 | 56 | 57 | WSW | SSW | | cloudy | fine |
| 10 | | | 59 | 64 | 55 | .63 | .55 | 57 | 56 | W | SW | | fine | cloudy |
| 11 | | | 58 | 65 | 59 | .57 | .47 | 55 | 60 | SSE | NE | fine | | |
| 12 | | .68 | 64 | 69 | 60 | .53 | .25 | 63 | 62 | SE | S | rain | cloudy | |
| 13 | ○ | | 66 | 75 | 60 | .31 | .38 | 60 | 58 | SSW | WSW | cloudy | | rain |
| 14 | | .12 | 68 | 69 | 58 | .45 | .56 | 55 | 54 | WSW | WSW | | fine | fine |
| 15 | | | 66 | 68 | 55 | .75 | .77 | 54 | 54 | W | WSW | fine | | |
| 16 | | .45 | 63 | 67 | 58 | .86 | .86 | 55 | 55 | WNW | WSW | | rain | |
| 17 | | | 64 | 70 | 60 | .88 | .90 | 54 | 53 | SW | W | | fine | |
| 18 | | | 68 | 73 | 61 | .90 | .86 | 53 | 52 | W | SW | | | |
| 19 | | | 68 | 73 | 62 | .84 | .84 | 52 | 53 | SW | NW | cloudy | | |

The quantity of Rain fallen in the month of May, was 1 inch and 71.100ths.

